# A computational tool suite to facilitate single-cell lineage tracing analyses

**DOI:** 10.1016/j.crmeth.2024.100780

**Published:** 2024-05-13

**Authors:** Joshua J. Waterfall, Adil Midoun, Leïla Perié

**Affiliations:** 1 Institut Curie, Université PSL, INSERM U830, Paris, France; 2 Institut Curie, Université PSL, Department of Translational Research, Paris, France; 3 Institut Curie, Université PSL, Sorbonne Université, CNRS UMR168, Laboratoire Physique de la Cellule et du Cancer, Paris, France

## Abstract

Tracking the lineage relationships of cell populations is of increasing interest in diverse biological contexts. In this issue of *Cell Reports Methods*, Holze et al. present a suite of computational tools to facilitate such analyses and encourage their broader application.

## Main text

Distinguishing how shared inheritance shapes cellular phenotypes is a central question in developmental biology, cancer heterogeneity, immunity, and many other biological fields. A powerful example of the utility of such lineage tracing experiments is the mapping of the developmental fate of every cell in the *Caenorhabditis elegans* embryo by direct observation via light microscopy.^1^ While classic techniques for lineage tracing, e.g., via Cre-mediated reporter expression or T cell receptor or B cell receptor diversification in lymphocytes have been used for decades, recent years have seen an explosion in new approaches.^2^^,^^3^ These newer approaches include the use of “endogenous” barcodes generated by somatic mutations in nuclear and mitochondrial genomes as well as a wide variety of exogenous barcoding methods leveraging different genetic engineering approaches such as CRISPR-Cas9, lentiviruses, terminal deoxynucleotidyl transferase (TdT), RAG 1 and 2 enzymes, and more. Importantly, these exogenous barcoding technologies work both in human cell culture/*ex vivo* systems as well as *in situ* for a variety of model organisms including mouse and zebrafish.

Coupled with simultaneous high-throughput molecular phenotyping of the cells, such as through single-cell RNA sequencing (scRNA-seq), these techniques are powerful molecular “microscopes” that can link the current state of a cell with its ancestral history.^3^ However, the introduction of PCR and sequencing errors and the unique aspects of each system pose substantial technical challenges for their analysis.^4^ Currently, researchers interested in adding lineage information to their favorite model system have no shortage of experimental protocols to choose from; however, there is a comparative lack of broadly applicable, flexible analysis tools capable of analyzing data from diverse protocols.^4^ Publishing in *Cell Reports Methods*, Holze et al.^5^ have developed a tool suite that complements existing tools^6^^,^^7^^,^^8^^,^^9^ and could greatly enhance the widespread adoption of such techniques by non-specialist teams. It also helps to address the crucial need for clearer guidelines and standardized metrics to evaluate new datasets, improving the overall reproducibility of single-cell lineage tracing results.^4^

**Figure 1:**
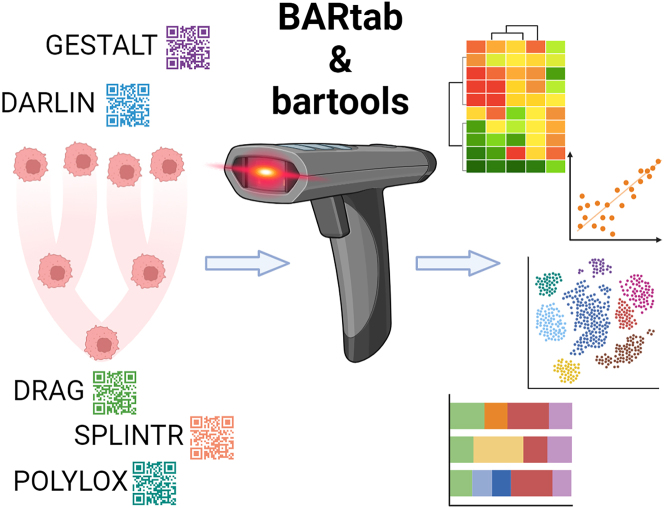
Schematic of BARtab and bartools applications Cell lineage can be determined by multiple experimental barcoding protocols (left). BARtab and bartools, published by Holze et al.^5^ in this issue of *Cell Reports Methods*, provide flexible, broadly applicable computational analysis tools that can analyze diverse barcoding experimental protocols, providing quality control assessment, diverse visualization tools, and statistical testing. Figure created using BioRender.com.

The BARtab and bartools analysis suite reported by Holze et al.^5^ is a significant step forward by simplifying single-cell lineage tracing results analysis for a broad community. Implemented both as a Nextflow pipeline and R package, these tools allow flexible analyses of diverse barcoding approaches with a particular focus on quality control, barcode identification and quantification, and multiple plotting options as well as statistical evaluation of barcode composition and diversity (Figure 1). The first tool, BARtab, is dedicated to early processing steps of fastq or bam files to identify and quantify barcodes. This can be applied both to bulk sequencing protocols as well as to single-cell protocols. Together with the CellBarcode R tool recently released,^6^ these are the first tools incorporating single-cell input from a diversity of wet lab protocols with high flexibility in both the design and lengths of the barcodes. Therefore, they could be broadly taken up by the community for standard analyses of diverse experimental approaches.

The second package, bartools, is dedicated to the further analysis of quantified barcode abundance estimates and can import both the BARtab output as well as results from other software packages in simple csv format. Bartools focuses specifically on barcode normalization, visualization, and evaluation of barcode composition and diversity alongside basic statistical testing. The introduction of intuitive visualization techniques and accompanying statistics greatly aids exploratory analysis of such large datasets and rapid QC evaluation of new experiments. For non-specialist teams, this will enable the incorporation of such approaches in their experimental toolbox. bartools does require the user to be familiar with the R environment, while other tools have provided Rshiny apps for even easier access.^8^^,^^9^ The authors describe extensive testing against previously published datasets, both from their own lab as well as others. This includes not only barcode assays performed by bulk sequencing as well as scRNA-seq but also spatial transcriptomics, demonstrating the most up-to-date range of utility.

The field faces upcoming challenges in providing guidelines on how to identify barcodes from noise and provide accurate barcode identification and quantification.^4^ It is easy to generate “normal looking” data that are in fact polluted by technical artifacts such as PCR or sequencing errors. Simulation tools incorporated into analysis suites have already started to address such issues,^6^ but more diverse barcode designs as well as biological and technical parameters need to be added. Additionally, such tools need to be extended to incorporate evolvable barcodes that introduce added complexity to the analysis.^7^ While evolvable barcodes have their own diversity of designs, incorporating them would provide a truly flexible suite of tools for all single-cell lineage analysis. Finally, maintaining and updating the existing tools over time is often a challenge in academia. The successful examples of Seurat and scanpy for scRNA-seq analyses should guide the field.
